# Multiscale analysis and optimal glioma therapeutic candidate discovery using the CANDO platform

**DOI:** 10.1186/s13321-026-01191-9

**Published:** 2026-04-12

**Authors:** Sumei Xu, Yakun Hu, William Mangione, Melissa Van Norden, Katherine Elefteriou, Zackary Falls, Ram Samudrala

**Affiliations:** 1 Phase I Clinical Trial Center, Xiangya Hospital, Central South University, 87 Xiangya Rd, Changsha, 410008 Hunan China; 2 National Clinical Research Center for Geriatric Disorders, Xiangya Hospital, Central South University, 87 Xiangya Rd, Changsha, 410008 Hunan China; 3 Department of Biomedical Informatics, University at Buffalo, 77 Goodell Street, Buffalo, NY 14203 USA

**Keywords:** Glioma, Multiscale drug discovery, Computational drug repurposing, Translational bioinformatics, Deep learning, Systems biology

## Abstract

Glioma is a highly malignant brain tumor with limited treatment options. We employed the Computational Analysis of Novel Drug Opportunities (CANDO) platform for multiscale therapeutic discovery to predict new glioma therapies. We began by computing interaction scores between extensive libraries of drugs/compounds and proteins to generate “interaction signatures” that model compound behavior on a proteomic scale. Compounds with signatures most similar to those of drugs approved for a given indication were considered potential treatments. These compounds were further ranked by degree of consensus in corresponding similarity lists. We benchmarked performance by measuring the recovery of approved drugs in these similarity and consensus lists at various cutoffs, using multiple metrics and comparing results to random controls and performance across all indications. Compounds ranked highly by consensus but not previously associated with the indication of interest were considered new predictions. Our benchmarking results showed that CANDO improved accuracy in identifying glioma-associated drugs across all cutoffs compared to random controls. Our predictions, supported by literature-based analysis, identified 24 potential glioma treatments, including approved drugs like vitamin D, taxanes, vinca alkaloids, topoisomerase inhibitors, and folic acid, as well as investigational compounds such as ginsenosides, chrysin, resiniferatoxin, and cryptotanshinone. Further functional annotation-based analysis of the top targets with the strongest interactions to these predictions identified Vitamin D3 receptor, thyroid hormone receptor, acetylcholinesterase, cyclin-dependent kinase 2, tubulin alpha chain, dihydrofolate reductase, and thymidylate synthase. These findings indicate that CANDO’s multitarget, multiscale framework is effective in identifying glioma drug candidates thereby informing new strategies for improving treatment.

**Scientific contribution**

(1) We present a robust, multiscale drug discovery framework that accurately recovers known glioma therapies and uncovers 24 novel candidates with strong literature and mechanistic support. (2) By modeling compound behavior across the proteome, our method pinpoints key targets–including VDR, CDK2, and DHFR–implicated in glioma biology. (3) This work positions CANDO as a powerful tool for rational repurposing and discovery of urgently needed treatments for aggressive brain tumors.

**Supplementary Information:**

The online version contains supplementary material available at 10.1186/s13321-026-01191-9.

## Introduction

Glioma is one of the most aggressive and fatal forms of malignant brain tumors, particularly prevalent among the elderly, with high rates of occurrence and mortality [1, 2]. Currently, chemotherapy is the primary treatment for glioma due to its aggressive progression, various pathologies, and the challenges associated with complete surgical removal [3, 4]. However, the effectiveness of chemotherapy is significantly limited by factors such as the selective permeability of the blood-brain barrier (BBB), neurotoxicity, and inadequate drug delivery to the tumor site [5–9]. Furthermore, the ATP-dependent efflux transporter, P-glycoprotein (P-gp), located on the BBB, contributes to the removal of chemotherapeutic agents [10]. A substantial proportion of patients with glioma (about 90%) experience tumor recurrence in the local area after initial treatment [11]. Unfortunately, effective therapeutic options for recurrent glioma are lacking. As a result, there is an urgent need to advance our understanding of the molecular pathology of glioma, identify new therapeutic targets, and develop innovative treatment strategies. A major challenge in modern medicine is the limited availability of new glioma drugs that can cross the BBB [12–14].

The process of drug discovery aims to identify chemical compounds with therapeutic potential for treating human diseases. Despite substantial advances, the success rate for the introduction of new drugs to the market has declined, with the average drug discovery pipeline now exceeding 12 years and costing more than two billion dollars [15, 16]. Computational approaches, such as virtual high-throughput screening, are increasingly being used to identify potential lead compounds by simulating and evaluating the binding affinity of numerous compounds against a target [17–20]. Challenges such as the vast combinatorial space of binding poses [21, 22] and ligand conformations [23, 24], coupled with the complex dynamics of these systems [25], limit the effectiveness of traditional virtual screening in reliably producing effective leads. Some computational methods stand out for their efficiency, accuracy, comprehensive assessment of interaction spaces, and broad exploration of chemical space, helping to address the limitations of conventional approaches [26–33]. Although computational screenings often focus on a single protein target, drugs in humans interact with various biological targets through processes such as absorption, distribution, metabolism, and excretion (ADME), influencing their efficacy and safety [31–37]. Considering drug interactions on a proteomic scale could yield more accurate predictions of bioactivity and safety by accounting for both primary and secondary targets, essential for optimizing therapeutic impact and minimizing toxicity.

We developed the Computational Analysis of Novel Drug Repurposing Opportunities (CANDO) platform for multitarget drug discovery, repurposing, and design, aiming to address the limitations of traditional single target, single disease approaches [38–53]. CANDO exploits the fact that drugs with regulatory approval for any clinical indication achieve therapeutic effects and favorable ADME through interactions with multiple biological targets, while off-target interactions are modulated to minimize adverse reactions. Hereafter, we refer to these as approved drugs. CANDO capitalizes on this inherent multitargeting property of small molecules by constructing interaction signatures that reflect drug/compound behaviors across various biological scales. The platform predicts putative drug candidates for every indication/disease by comparing and ranking these interaction signatures in an all-against-all manner, with the hypothesis that drugs/compounds with similar interaction signatures are more likely to display similar biological behavior. The platform is benchmarked by evaluating the recovery of known drug-indication associations, drugs previously reported in the literature to be linked to glioma-related mechanisms, in these ranked lists of interaction signatures within specified cutoffs. CANDO therefore deepens our understanding of small molecule therapeutics and their effects on proteins, pathways, and various diseases by leveraging vast multiscale biomedical data on biological systems and the phenotypic impact of their modulation. In addition to rigorous benchmarking [38–53], CANDO and/or its components have been extensively validated prospectively in the context of more than a dozen indications [38, 41, 47, 50–52, 54–64]. Herein, we describe the use of CANDO to predict novel drug candidates for glioma treatment. This study benchmarks the CANDO platform for glioma and identifies both rediscovered and novel candidate compounds, rather than introducing new algorithmic or cheminformatic methods.

## Methods

### Applying the CANDO platform for glioma drug discovery overview

**Fig. 1:**
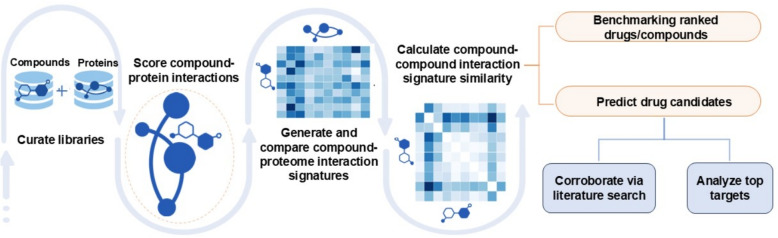
Overview of the primary pipeline for generating novel putative drug candidate predictions for glioma within the CANDO multiscale drug discovery platform. Interaction scores between every protein and drug/compound in the corresponding libraries were calculated using our bioanalytical docking protocol (BANDOCK) [38–53]. This resulted in a compound-proteome interaction signature for each drug/compound describing its functional behavior. Interaction signature similarity was then calculated by comparing pairs of drug-proteome interaction signatures in an all-against-all manner. These interaction signature similarities were sorted and ranked for all drugs approved for an indication and used to benchmark performance and generate putative drug candidates. Benchmarking was conducted by measuring how approved drugs were recovered at various cutoffs. We performed a literature-based analysis to corroborate the glioma drug candidates for their potential to treat this disease. Finally, we identified the protein targets with the highest predicted likelihood of binding with these candidates and further corroborated them using protein functional annotations. The CANDO platform successfully identified multiple candidates demonstrating significant anti-glioma potential, offering a promising avenue to address the current lack of effective treatments for this disease

We developed a pipeline within the CANDO platform to identify potential drug candidates for glioma (Fig. 1). Our approach is based on the hypothesis that drugs/compounds with similar interactions across entire proteomes (“interaction signatures”) are more likely to share therapeutic effects. Signatures were generated by calculating interaction scores between every drug/compound and a comprehensive library of proteins to capture the proteome-wide behaviors of a compound. Compounds with interaction signatures closely matching those known associations were identified as potential treatments. We benchmarked performance by measuring how frequently known associations for a given indication were retrieved at various cutoffs in ranked lists of predictions. Next, we compared our glioma-specific results against random controls, as well as across all indications. The novel predictions for glioma were then corroborated through literature-based analysis to identify the highest confidence drug candidates. Finally, we conducted a consensus analysis of proteins with the strongest interactions to these novel glioma drug candidates which was further corroborated using protein functional annotations.

### Curating compound/protein libraries and indication mapping

Our drug/compound library, sourced mainly from DrugBank [65], comprises 2,449 approved drugs and 10,741 experimental or investigational compounds, totaling 13,457 molecules. The “Homo sapiens AlphaFold2” (AF2) protein library was curated following the application of the AlphaFold2 structure prediction program [66] to the Homo sapiens proteome, yielding 20,295 proteins used for this study. The Comparative Toxicogenomics Database (CTD) was used to map the 2,449 approved drugs to 22,771 drug-indication associations based on DrugBank identifiers for drugs and compounds and Medical Subject Headings (MeSH) terms for approved/associated indications [67, 68]. Benchmarking, which uses a leave-one-out approach ("Benchmarking" section), was carried out on indications with at least two approved drugs, yielding a drug-indication mapping consisting of 1,595 indications and 13,226 associations. There were 35 known associations in our drug-indication mapping for the indication glioma (MeSH identifier: D005910).

### Scoring compound-protein interactions and generating interaction signatures

Interaction scores between each compound and protein were computed using our in-house bioanalytical docking protocol (BANDOCK); these scores serve as a proxy for binding strength/probability [38, 39, 41, 43, 46, 48]. Binding site prediction was first performed using the COACH algorithm from the I-TASSER suite (version 5.1) [69]. COACH utilizes a library of protein structures bound to ligands, determined through x-ray diffraction, to predict the binding sites and corresponding ligands for each protein based on structural and sequential similarity [70]. BANDOCK then calculates interaction scores by comparing the COACH predicted ligands to the query compound, using similarity between their Extended Connectivity Fingerprints with a diameter of 4 (ECFP4), generated via RDKit [71]. The chemical similarity score is quantified using the Sorenson-Dice coefficient [72], which reflects the similarity between the query compound and the predicted ligand. The highest chemical similarity score is multiplied by the corresponding COACH binding site confidence score to assign an interaction score between a compound and a protein by BANDOCK [38, 39, 41, 43, 46, 48]. BANDOCK is applied between every compound and all proteins in the library, producing compound-proteome interaction signatures describing (compound) behavior.

### Calculating ranked compound similarity lists

CANDO calculates all-against-all similarities between compound-proteome interaction signatures to compute drug repurposing accuracy and predict drug candidates [46]. We employed cosine distance for similarity calculations instead of the usual root-mean-square deviation (RMSD) [53] as it enhanced computational speed while maintaining performance. This process was repeated iteratively for all compound pairs in the library, producing a ranked similarity list for each compound.

### Benchmarking

Compounds are ranked by the number of times they appear in the similarity lists of the associated drugs above a certain cutoff, resulting in a consensus list. We benchmarked the performance of CANDO by evaluating the recovery of known associations within similarity lists and aggregated consensus lists across various cutoffs using multiple metrics. The consensus lists classify/rank compounds according to their consensus scores, which reflect how frequently they appear in multiple similarity lists corresponding to all approved drugs for an indication. As mentioned above ("Curating compound/protein libraries and indication mapping" section), we used drug-indication mappings from the Comparative Toxicogenomics Database (CTD) [73] to determine the ranking of approved drugs within specific cutoffs (e.g., top 10, 25, 50, 100) in the similarity and consensus lists of drugs for a given indication with at least two approved drugs [38–53]. Benchmarking performance for all indications, including glioma, was compared to a random control that calculated the probability of correctly selecting an approved drug for an indication by chance, using a hypergeometric distribution [51, 74].

CANDO calculates the following metrics developed in-house to assess performance: indication accuracy, average indication accuracy, new indication accuracy, and new average indication accuracy. Indication accuracy (IA) is the percentage of cases in which at least one approved drug for a given indication appears within a specified rank cutoff in the similarity list of another drug associated with that same indication. Averaging the IA values for all indications with at least two approved drugs produces the average indication accuracy (AIA). New indication accuracy (nIA) captures the frequency with which approved drugs for a given indication appear within particular cutoffs in the consensus list for that indication. The nIA is averaged across all indications to yield the new average indication accuracy (nAIA) metric.

CANDO also calculates the normalized discounted cumulative gain (NDCG) metric, an evaluation measure commonly used in information retrieval to assess the relevance of ranked items based on their positions [75, 76], to evaluate our predictions. In CANDO, NDCG evaluates how effectively a given pipeline prioritizes approved drugs for a specific indication within its similarity lists at specified cutoffs. The NDCG score ranges from 0 to 1, with 1 indicating a perfect ranking [51]. Similarly, the new NDCG (nNDCG) metric assesses the recovery of approved drugs across specified cutoffs in the consensus list for an indication.

In addition to benchmarking against random controls, we implemented a 2D ligand-based similarity pipeline alongside the proteomic-signature-based benchmarking to contextualize our approach against a conventional and computationally inexpensive baseline. For each approved drug in the DrugBank-derived library, 2D chemical structures were parsed into molecular graph objects from structure files, and circular substructure fingerprints (ECFP4; radius 2) were generated as fixed-length bit vectors encoding the presence or absence of local chemical environments. Pairwise ligand similarities were then computed using the Tanimoto coefficient. This constituted the unfiltered Tanimoto drug-drug matrix.

We hypothesized that the strong performance of the unfiltered ECFP4/Tanimoto pipeline could be influenced by common near-duplicate “me too” drug relationships, which are common among approved and investigational compounds [77–80]. To assess the impact of these close analogs, we created a filtered Tanimoto matrix using a per-drug uniqueness filter to emphasize structurally unique relationships and prevent near-duplicate analogs from dominating recovery. For each query drug, we set the highest two percent of Tanimoto similarities in its similarity list to zero so those pairs did not contribute to the ranking. This percentile filter corresponds to a Tanimoto score of roughly 0.2 as the distribution was very skewed. Each drug’s ranked similarity list was produced by sorting all other drugs by decreasing similarity. These similarity lists were benchmarked using the same leave-one-out framework, cutoffs, and metric definitions used for the proteomic pipelines, enabling a direct comparison between proteomic signatures and standard ligand-only baselines within the same dataset and evaluation protocol.

We evaluated several fusion pipelines that combine the proteomic and unfiltered Tanimoto pipelines. The fusion pipelines were constructed by generating a fused neighbor ranking from the two input pipelines’ similarity lists by combining either their ranks or their scores for each query drug. We evaluated multiple operators, including the minimum, maximum, average, sum and product of ranks; the product of similarity scores; and the product of distances. The best-performing approach was the product of similarity scores, computed by converting each pipeline’s distances to similarities (similarity = 1−distance), multiplying the two similarities, and converting the result back to a distance-like value (distance = 1−fused_similarity). This was the only fusion strategy that consistently outperformed the unfiltered Tanimoto pipeline across our benchmarks and thus the only one included in our results; the minimum-rank fusion pipeline performed comparably to the unfiltered Tanimoto pipeline but did not exceed it. We also explored fusions between the proteomic pipeline and a filtered Tanimoto pipeline, but these combinations did not surpass their input pipelines as reliably as the aforementioned similarity-score product fusion, and we therefore omit them from the final results.

### Generating drug predictions and corroborating them using literature searches

The CANDO platform was applied to predict novel putative therapeutics for glioma (MeSH identifier: D005910) which had 35 known associations in our drug-indication mapping ("Curating compound/protein libraries and indication mapping" section). As described above, drugs/compounds with interaction signatures similar to those of drugs associated with glioma were ranked. Next, their frequency, or consensus, among the similarity lists was used to identify the top 100 novel drug candidates for glioma. A binomial cumulative density function was used to calculate the probability of the observed frequency of occurrence of each drug among the top 100 ranked compounds in similarity lists under random ranking. We conducted a literature review using search terms that consisted of the name of each putative drug candidate and “glioma” in Google Scholar and PubMed. We categorized the candidates as follows: *high-corroboration* for compounds supported by two or more studies showing positive glioma treatment results, referred to hereafter as corroborated candidates; *low-corroboration* for compounds targeting glioma-related pathways or supported by a single positive study but lacking confirmation; and *no data found* when no data was present to arrive at any conclusion regarding corroboration. Each drug was assigned to a drug class using DrugBank annotations where available; compounds without a DrugBank class assignment were labeled unclassified.

### Analyzing top targets and associated pathways for glioma

We used our in-house top targets protocol to identify the proteins with the strongest interactions with each putative drug candidate that was classified as high-corroboration above. Interaction scores were calculated as described previously ("Scoring compound-protein interactions and generating interaction signatures" section) using the BANDOCK protocol, where higher scores (maximum of 1.0) indicate stronger predicted interactions. We then conducted a literature search on Google Scholar and PubMed with the names of the putative drug candidates and proteins to find corroborative evidence supporting the target rationale used by CANDO in generating predictions. We used this information to analyze whether the top targets of the putative drug candidates overlap with proteins in biochemical pathways linked to glioma.

### Assessing corroboration between protein functional annotations and predicted top targets

We curated three protein libraries, or “gold standards”, from UniProt [81], GeneCards [82], and a comprehensive literature search to serve as references for evaluating the target predictions for putative glioma treatment candidates. The literature search, data presented in Table 2, focused on identifying targets implicated in glioma from the top targets analysis for corroborated putative drug candidates generated by the CANDO platform. The benchmarks assessed the overlap between the gold standard libraries and the top protein targets predicted by CANDO for the top 24 drug candidate predictions. This assessment was repeated with a random set of 24 drug predictions and the bottom 24 drug predictions as controls. The bottom 24 drug predictions were filtered to include only compounds with at least five heavy atoms to maintain meaningful molecular complexity. To quantify the alignment between the gold standards and the predictions, we employed three key metrics: (A) frequency distribution, (B) percentage overlap, and (C) the Jaccard coefficient, a commonly used metric for assessing similarity across datasets [83, 84]. The Jaccard coefficient calculates the ratio of the intersection and union of two groups and is defined as:

$$ J(A, B) = \frac{|A \cap B|}{|A \cup B|} $$

In this context, *A* represents proteins annotated with glioma-related functions, and *B* represents the top protein targets predicted by CANDO. A high Jaccard coefficient indicates that CANDO accurately identifies protein targets that are independently corroborated by functional annotation libraries.

Additionally, we compared the Jaccard coefficient across glioma and other disease indications by using the top predicted targets from the top 24 or top 100 drug candidate predictions for glioma as one group, and functional protein annotations from UniProt as the other group. Selected indications included cancer indications (e.g., metastatic melanoma, non-small cell lung cancer, acute myeloid leukemia) and non-cancer diseases (e.g., Alzheimer’s disease, rheumatoid arthritis, asthma). Additionally, we analyzed functional annotations for protein targets in the UniProt database to assess their association with glioma and other disease indications. The Jaccard coefficient was computed separately for glioma-related targets and targets associated with other diseases, including cancer indications (e.g., metastatic melanoma, non-small cell lung cancer, acute myeloid leukemia) and non-cancer diseases (e.g., Alzheimer’s disease, rheumatoid arthritis, asthma). The comparison involved the predicted targets from the top 24 and top 100 drug candidate predictions to evaluate performance differences across indications.

## Results

In summary, the results of this study provided strong evidence for the utility of the CANDO platform in identifying putative drug candidates for glioma. The multitarget approach enabled precise ranking and identification of compounds based on their interaction signatures across the human proteome for treating glioma. The drug candidates exhibited high interaction signature similarity to those of established glioma treatments and were observed to target critical pathways associated with glioma pathogenesis. Benchmarking against random controls and an ECFP4/Tanimoto fingerprint baseline further supported the robustness of the platform, with the proteomic signature pipeline showing strong performance.

### Benchmarking performance

**Fig. 2:**
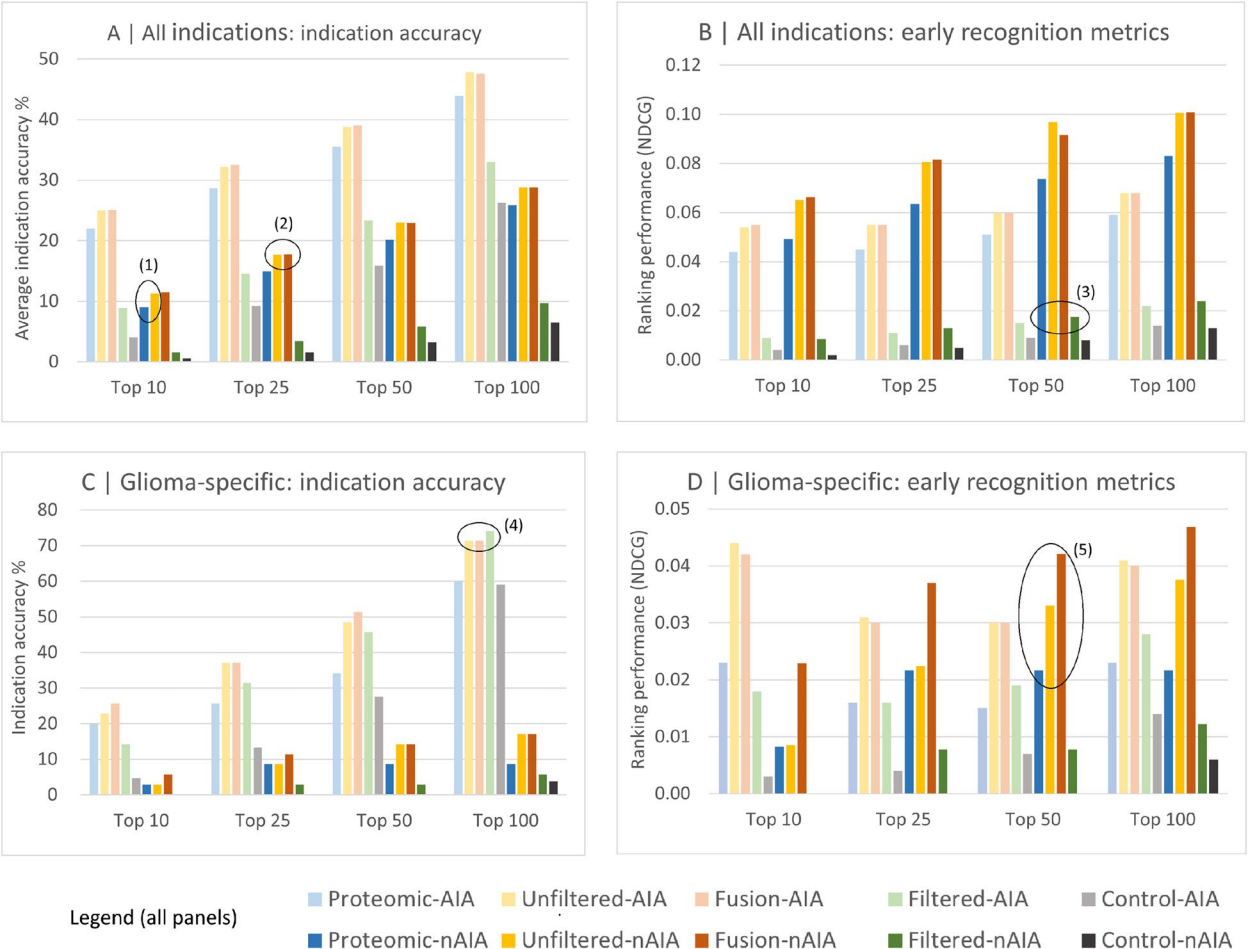
Benchmarking performance of the CANDO platform comparing proteomic, unfiltered ECFP4/Tanimoto, fusion, and filtered Tanimoto pipelines, as well as control. Panels **A**, **B** summarize results across all indications with at least two approved drugs, while panels **C**, **D** focus specifically on glioma. Applying a uniqueness filter to the fingerprinting pipeline (ECFP4/Tanimoto) to suppress highly prevalent, near-duplicate “me too” drug relationships ("Benchmarking" Section) attenuates its advantage relative to the proteomic pipeline (1), reducing fingerprinting performance to below that of the other three pipelines for most metrics (3), except for (4) glioma-specific IA and glioma-specific NDCG [77–80]. The proteomic pipeline remains vital for generating predictions as it uses mechanistic rather than chemical similarity, with the latter likely to turn up close analogs. (2) and (5) The fusion pipeline ("Benchmarking" Section) performs better than the component proteomic or fingerprinting pipelines for most consensus metrics. CANDO consistently outperforms random controls across all cutoffs and metric groups. For example, at the top 100 cutoff across all indications, the proteomic pipeline AIA is approximately two folds better than the random control, while NDCG and nNDCG values are four to ten folds better than controls. Glioma-specific benchmarking shows similar separation from random controls across both metric groups, indicating robust recovery and prioritization of known associations. Exact benchmarking values for all metrics and cutoffs are reported in the Supplementary Table S1

Figure 2 summarizes the benchmarking performance of the CANDO proteomic signature pipeline for all indications and glioma specifically relative to the fingerprinting, fusion, and fingerprinting filtered pipelines as well as random controls, evaluated using both similarity and consensus lists ("Benchmarking" Section). The approved drug library included 35 known drugs associated with glioma. Benchmarking was performed across four cutoffs, with exact metric values reported in Supplementary Table S1.

While the unfiltered fingerprinting (ECFP4/Tanimoto) pipeline performs better than the proteomic signature pipeline across all cutoffs, applying a uniqueness filter that suppresses highly prevalent, near-duplicate “me too” drugs reduces the advantage conferred by these close analogs and improves the relative standing of the proteomic pipeline. The filtered ECFP4/Tanimoto ligand-similarity pipeline, which excludes the highest-similarity relationships to de-emphasize “me too” neighbors, performs markedly worse than the proteomic and the other two CANDO pipelines across all-indications metrics. In new average indication accuracy, it performs nearly sixfold worse than the proteomic and tenfold worse than the unfiltered Tanimoto and fusion pipelines at the Top 10 cutoff. However, its performance is proportionally closer on glioma-specific metrics, falling within 10% of the fusion and unfiltered fingerprinting pipelines at the Top 25 cutoff for indication accuracy and even surpassing the other three pipelines at the Top 100 cutoff. For the consensus-based metrics (nIA, nNDCG), this narrowing of the gap from all-indications to glioma-specific benchmarking is less pronounced. By contrast, the unfiltered fingerprinting pipeline outperforms the proteomic signature pipeline by a margin of 5–15% on accuracy-based metrics and by 20–200% on early-recognition metrics, with the difference being larger on all-indications benchmarking.

The fusion pipeline, a multiplication of proteomic and unfiltered Tanimoto similarity scores, closely matches the unfiltered Tanimoto score across the accuracy-based metrics and more frequently outperforms it than underperforms it, supporting complementarity between proteomic and ligand-derived similarity signals. It also performs markedly better on glioma-specific nNDCG than either the proteomic or Tanimoto pipeline, achieving an over twofold improvement at the top-10 cutoff.

Across all indications with at least two approved drugs, the four CANDO pipelines consistently outperform random controls for both accuracy-based and early-recognition-based metrics (Fig. 2A, B). Accuracy-based metrics increase monotonically with less stringent cutoffs and show substantial separation from random controls across all cutoffs. For example, at the top 100 cutoff, average indication accuracy is approximately two folds higher than the random control. Early-recognition-based metrics exhibit similar behavior, with NDCG and nNDCG values several folds higher than controls, indicating improved prioritization of relevant drugs throughout the ranked lists.

Glioma-specific benchmarking demonstrates comparable trends (Fig. 2C, D). Accuracy-based metrics show strong recovery of known glioma-associated drugs across all cutoffs, with particularly clear separation from random controls at more stringent cutoffs. Ranking-based evaluation further confirms that glioma-relevant drugs are prioritized more effectively than expected by chance, with NDCG and nNDCG values consistently exceeding those of random controls (Supplementary Table S1).

**Table 1:** Predicted drug candidates for glioma using CANDO platform that were corroborated using literature analysis

Drug rank	Consensus score	Average score	Probability	Drug name	Drug class
1	4	28.5	2.68E-05	Calcifediol	Vitamin D analog
2	4	30.2	2.68E-05	Ergocalciferol	Vitamin D analog
3	4	37.5	2.68E-05	Cholecalciferol	Vitamin D analog
6	3	9.0	4.22E-04	Cabazitaxel	Taxanes
10	3	10.3	4.22E-04	Docetaxel	Taxanes
25	3	22.0	4.22E-04	Calcitriol	Vitamin D analog
26	3	22.3	4.22E-04	Tacalcitol	Vitamin D analog
29	3	23.0	4.22E-04	Vinflunine	Vinca alkaloids
31	3	23.3	4.22E-04	Vinorelbine	Vinca alkaloids
32	3	23.3	4.22E-04	Folic acid	Glutamic acid derivative
49	3	32.7	4.22E-04	Topotecan	Topoisomerase I inhibitors
50	3	33.0	4.22E-04	Calcipotriol	Vitamin D analog
51	3	35.3	4.22E-04	Ergosterol	Vitamin D analog
55	3	38.3	4.22E-04	Vinblastine	Vinca alkaloids
56	3	38.3	4.22E-04	Lanosterol	Triterpene
63	3	44.0	4.22E-04	Brivaracetam	Alpha amino acid derivative
64	3	44.3	4.22E-04	Loperamide	Diphenylmethane
65	3	44.3	4.22E-04	Ginsenosides	Unclassified
66	3	47.0	4.22E-04	Gimatecan	Topoisomerase I inhibitors
73	3	49.7	4.22E-04	Ortataxel	Tetracarboxylic acid
74	3	50.0	4.22E-04	Resiniferatoxin	Capsaicin analog
78	3	51.7	4.22E-04	Irinotecan	Topoisomerase I inhibitors
80	3	52.7	4.22E-04	Chrysin	Unclassified
95	3	66.0	4.22E-04	Cryptotanshinone	Unclassified

The names of the 24 corroborated drug candidates ("Identifying drug candidates" section), along with their ranks, consensus/average scores, and probability values are listed. The consensus score represents the number of drug–drug interaction signature similarity lists in which a compound appeared within a particular cutoff. The probability estimates the likelihood of a particular ranked compound appearing by chance, with lower values indicating a better outcome. The overall ranking of a potential drug is determined first by its consensus score and then by its average rank ("Generating drug predictions and corroborating them using literature searches" section). The best ranked compounds in this consensus list are considered to be the top predictions for an indication. Vitamin D includes a group of compounds such as calcifediol, ergocalciferol, and cholecalciferol, which are ranked as the top three predictions with highest consensus score. This analysis indicates that the signature similarity pipeline within the CANDO platform can generate putative drug candidates for glioma

### Identifying drug candidates

We used the CANDO platform to predict potential drug candidates for glioma ("Generating drug predictions and corroborating them using literature searches" section). The 24 most compelling corroborated predictions based on ranking metrics from the platform and literature analysis are shown in Table 1. The list of all the top 100 putative drug candidates is given in Supplementary Table S2. The top ranked drug candidates were vitamin D compounds: calcifediol, ergocalciferol, and cholecalciferol. Additional drug candidates for glioma included taxanes (cabazitaxel), vinca alkaloids (vinflunine), and topoisomerase inhibitors (topotecan).

### Analyzing targets and pathways related to glioma

**Fig. 3:**
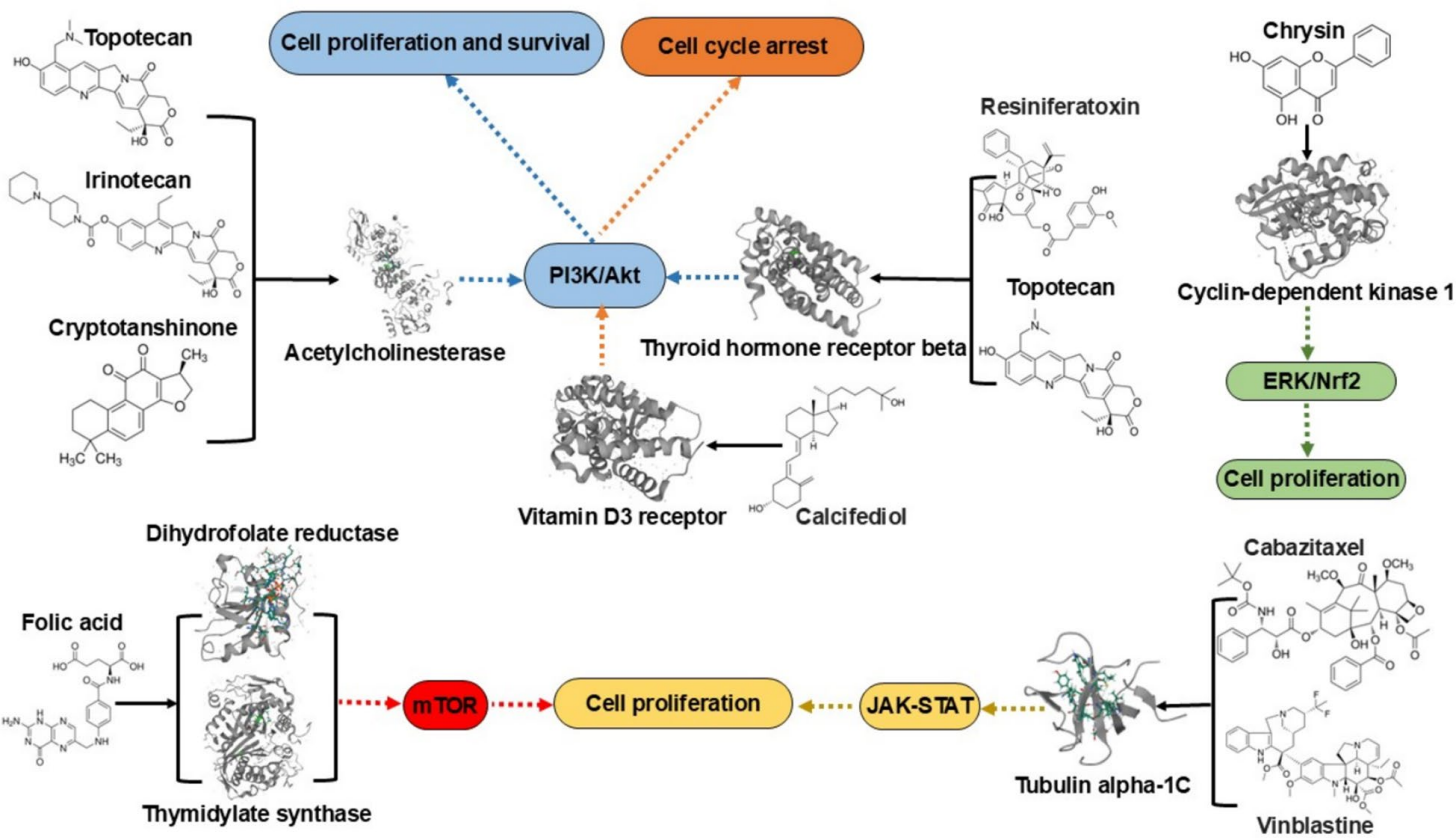
Downstream pathways of top targets for putative drugs for glioma treatment predicted by CANDO. The top targets for putative drugs for glioma are those with the strongest interactions as predicted by our CANDO platform (Table 2) and verified by a functional annotation search ("Determining overlap between protein functional annotations and CANDO predicted top targets" section). The phosphatidylinositol-3’-kinase (PI3K)/Akt, mammalian target of rapamycin (mTOR), Janus kinase (JAK)/signal transducer and activator of transcription 3 (STAT3) pathways play important roles in the biology of malignant gliomas [85–88]. Topotecan, irinotecan, and cryptotanshinone all interact with acetylcholinesterase (ACHE), ranking 8th (topotecan and irinotecan) and 1st (cryptotanshinone), respectively. Compounds resiniferatoxin and topotecan strongly interact with the thyroid hormone receptor beta (THRB). The targets ACHE and THRB both influence glioma cell proliferation and survival by regulating the PI3K/Akt signaling pathway (blue). Cell cycle arrest is one of the most well-studied mechanisms accounting for the antitumor activity of vitamin D in gliomas (orange). The compound chrysin interacts with cyclin-dependent kinase 1 (CDK1), targeting glioma cell proliferation via the ERK/Nrf2 signaling pathway (green). Dihydrofolate reductase (DHFR) and thymidylate synthase (TYMS) are key targets of folic acid, modulating glioma cell proliferation through the mTOR signaling pathway (red). Taxanes (e.g., cabazitaxel) and vinca alkaloids (e.g., vinblastine) interact with tubulin alpha-1C, influencing glioma through the JAK-STAT pathway (yellow). Our study allows for comprehensive mechanistic understanding of drug candidate behavior across multiple scales, showcasing the CANDO platform’s capability to accurately identify novel drug candidates and their mechanisms through a multifaceted strategy

The information considered when selecting putative drug candidates for novel treatment included the top (i.e., strongest interaction) protein targets predicted by CANDO, protein and pathway interactions corroborated using the literature, and studies of small molecules in the treatment of glioma observed in the literature "Analyzing top targets and associated pathways for glioma" section. The top targets predicted by CANDO are outlined in Table 2 and encompass Vitamin D3 receptor, thyroid hormone receptor, acetylcholinesterase, cyclin-dependent kinase 2, tubulin alpha chain, dihydrofolate reductase, and thymidylate synthase. Among these, the strongest interaction was observed between the Vitamin D3 receptor and calcifediol, with a BANDOCK score of 0.850. Figure 3 highlights various important related pathways implicated in the pathogenesis of glioma, including phosphatidylinositol-3’-kinase (PI3K)/Akt, mammalian target of rapamycin (mTOR), and Janus kinase (JAK)/signal transducer and activator of transcription (STAT) pathways.

### Determining overlap between protein functional annotations and CANDO predicted top targets

**Fig. 4:**
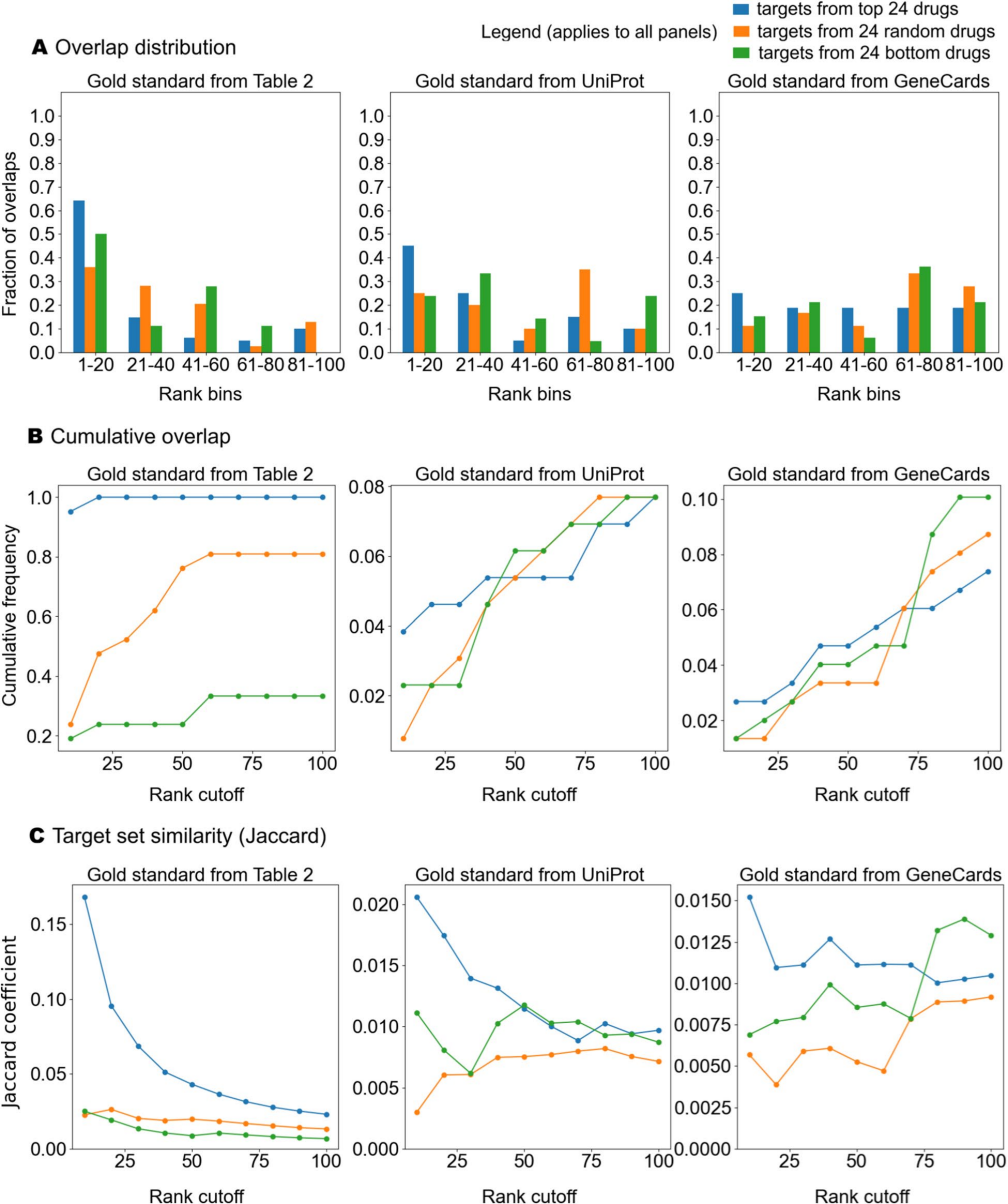
Overlap between protein functional annotations and CANDO predicted top targets across gold standard libraries. This figure compares the overlap between CANDO predicted targets and gold standard annotations (Table 2, UniProt, and GeneCards) for glioma-related proteins. **A** Frequency distributions show the proportion of predicted targets that overlap with gold standard proteins within rank bins for top 24 drug candidate predictions (blue), random 24 drug candidate predictions (orange), and bottom 24 drug candidate predictions (green). **B** The line graphs show the percentage of gold standard proteins that overlap with prediction targets as a function of rank cutoff (from 10 to 100). **C** Targets from the top 24 drug candidate predictions demonstrate a higher Jaccard coefficient compared to from random 24 and bottom 24 drug candidate predictions across all gold standards. The Jaccard coefficient quantifies the similarity between CANDO predicted targets and gold standard targets. Each column corresponds to a different gold standard: Table 2 (left), UniProt (center), and GeneCards (right). Results demonstrate targets from the top 24 drug candidate predictions generally reflect a stronger signal compared to that of random or bottom drug candidate predictions, highlighting the predictive accuracy of the top candidates

Figure 4A illustrates the frequency distribution of overlaps between our three gold standard protein libraries and the top protein targets predicted by CANDO. For all gold standards, targets of the top 24 drug candidates showed the highest proportion of overlaps within the highest ranked bin (1-20). In contrast, targets from the random 24 drug candidates and bottom 24 drug candidates exhibited a comparatively uniform distribution across the 5 bins. Figure 4B presents the cumulative percentage overlap as a function of rank cutoff for the predicted targets across the gold standard libraries. The targets from the top 24 drug candidate predictions demonstrated a near-saturation of overlap at lower rank cutoffs (e.g., 80% overlap by rank 20 for Table 2 and UniProt), emphasizing their strong alignment with gold standard targets. In contrast, the targets from the random 24 and bottom 24 drug candidate predictions exhibited a slower increase in overlap percentage, with cumulative overlaps remaining below 10% even at a rank cutoff of 100 for Table 2 and GeneCards. As shown in Fig. 4C, the Jaccard coefficient values further corroborate the findings from the frequency distribution and overlap percentage analyses. Across all libraries, the Jaccard coefficient for the top protein targets from the top 24 drug candidate predictions was consistently higher compared to those derived from the random 24 or bottom 24 drug candidate predictions.

**Fig. 5:**
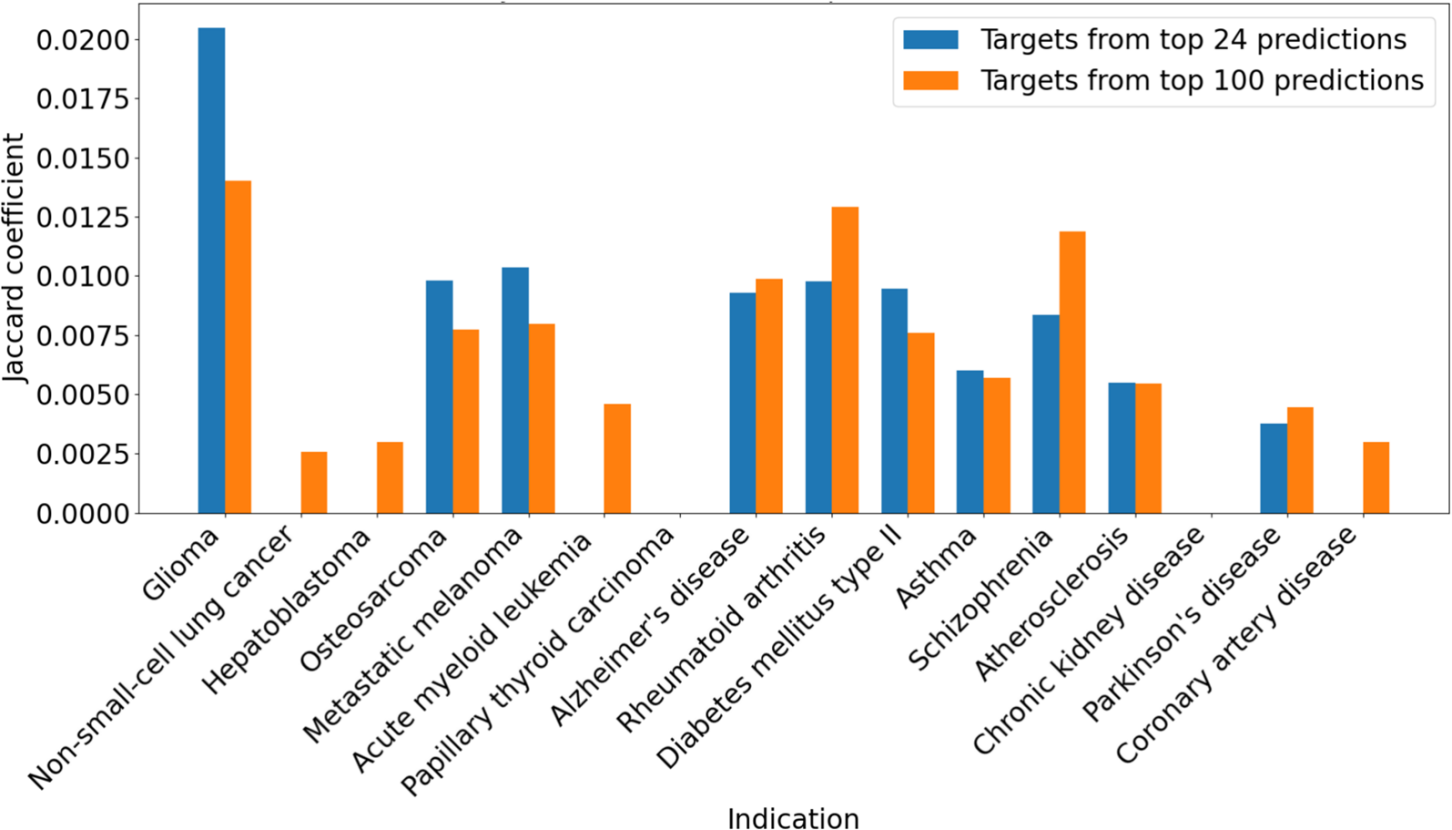
Overlap between protein functional annotations and CANDO predicted top targets across indications. This bar chart compares the overlap between targets with the strongest predicted interactions to our top drug candidates and existing protein annotations across glioma and other indications, using the Jaccard coefficient (vertical axes). The Jaccard coefficient quantifies the overlap between protein functional annotations (from UniProt) and CANDO-predicted drug targets. Two comparisons are made: the overlap with top 24 drug candidate predictions (blue) and the overlap with top 100 drug candidate predictions (orange). A higher coefficient indicates stronger alignment between the predicted and known targets. The horizontal axis lists the various indications, including cancers (glioma, hepatoblastoma, metastatic melanoma) and non-cancer conditions (diabetes mellitus type II, coronary artery disease, and chronic kidney disease). Results show that the Jaccard coefficient for glioma is notably higher than that of other indications, highlighting the effectiveness of the CANDO platform in identifying glioma-related protein targets

We found that the Jaccard coefficient for top (rank $\le 10$) predicted targets of the top 24 and top 100 drug candidate predictions for glioma was higher when compared to UniProt glioma protein functional annotations (Fig. 5). In contrast, the Jaccard coefficient was lower when comparing glioma targets to protein functional annotations for other indications. Indications demonstrating a lower Jaccard coefficient include other cancer indications such as non-small cell lung cancer and metastatic melanoma, as well as non-cancer diseases like Alzheimer’s disease and rheumatoid arthritis. This result suggests that the top predicted glioma targets identified by CANDO are more functionally relevant to glioma-related gold standard protein targets than those of other indications, highlighting the effectiveness of the pipeline in identifying meaningful targets. When compared to the broader rank distribution shown in the earlier line plot (Fig. 4), the rank $\le 10$ results highlight the ability of the pipeline to capture high-confidence and/or known targets for glioma. This trend underscores the utility of using stringent rank cutoffs to identify highly specific target overlaps, particularly for glioma.

**Table 2:** Top targets analysis for corroborated putative drug candidates for glioma generated by the CANDO platform

Drug rank	Drug name	Target rank	Target identifier	Score	Target name	Evidence
1	Calcifediol	1	P11473	0.850	Vitamin D3 receptor	Yuan et al. [89]
2	Ergocalciferol	32	P11473	0.605		Diesel et al. [90]
3	Cholecalciferol	3	P11473	0.740		Sze-Ching Lo et al. [91]
6	Cabazitaxel	13	P68363	0.725	Tubulin alpha-1B chain	Liu et al. [92] and Hu et al. [93]
		19	Q9BQE3	0.716	Tubulin alpha-1C chain	
10	Docetaxel	13	P68363	0.800	Tubulin alpha-1B chain	
		19	Q9BQE3	0.790	Tubulin alpha-1C chain	
73	Ortataxel	13	P68363	0.580	Tubulin alpha-1B chain	
		19	Q9BQE3	0.572	Tubulin alpha-1C chain	
29	Vinflunine	2	P68363	0.672	Tubulin alpha-1B chain	
		18	Q9BQE3	0.633	Tubulin alpha-1C chain	
31	Vinorelbine	2	P68363	0.669	Tubulin alpha-1B chain	
		18	Q9BQE3	0.631	Tubulin alpha-1C chain	
55	Vinblastine	2	P68363	0.765	Tubulin alpha-1B chain	
		18	Q9BQE3	0.721	Tubulin alpha-1C chain	
49	Topotecan	2	P11387	0.565	DNA topoisomerase I	Zhang et al. [94], Ma et al. [95], Zhang et al. [96], Li et al. [97], Obukhova et al. [98], Tsuji et al. [99], Jones et al. [100], Guo et al. [101], and Lei et al. [102]
		3	P06276	0.373	Cholinesterase	
		4	P10828	0.371	Thyroid hormone receptor beta	
		5	P02768	0.370	Albumin	
		8	P22303	0.359	Acetylcholinesterase	
		9	P27487	0.358	Dipeptidyl peptidase 4	
		97	O00763	0.301	Acetyl-CoA carboxylase 2	
66	Gimatecan	2	P11387	0.459	DNA topoisomerase I	
		3	P27487	0.392	Dipeptidyl peptidase 4	
		4	P14174	0.389	Macrophage migration inhibitory factor	
		7	P15559	0.373	NAD(P)H dehydrogenase quinone 1	
		10	O00763	0.364	Acetyl-CoA carboxylase 2	
78	Irinotecan	2	P11387	0.466	DNA topoisomerase I	
		8	P22303	0.390	Acetylcholinesterase	
		37	O00763	0.324	Acetyl-CoA carboxylase 2	
		47	P15559	0.317	NAD(P)H dehydrogenase quinone 1	
56	Lanosterol	1	P48449	0.960	Lanosterol synthase	Nguyen et al. [103], Han et al. [104], and Li et al. [97]
		9	Q9Y6A2	0.437	Cholesterol 24-hydroxylase	
		10	P02768	0.435	Albumin	
65	Ginsenosides	1	P48449	0.610	Lanosterol synthase	
		4	P02768	0.503	Albumin	
32	Folic acid	1	P00374	0.755	Dihydrofolate reductase	Zhao et al. [105], Kunikowska et al. [106], and Ding et al. [107]
		3	Q04609	0.723	Glutamate carboxypeptidase 2	
		6	P04818	0.688	Thymidylate synthase	
63	Brivaracetam	1	P24941	0.390	Cyclin-dependent kinase 2	Liu et al. [108]
64	Loperamide	2	Q9Y6A2	0.522	Cholesterol 24-hydroxylase	Han et al. [104], Was et al. [109], and Jones et al. [100]
		5	Q92769	0.467	Histone deacetylase 2	
		24	O00763	0.440	Acetyl-CoA carboxylase 2	
74	Resiniferatoxin	2	P10828	0.425	Thyroid hormone receptor beta	Zhang et al. [96] and Lu et al. [110]
		8	Q96GR4	0.402	Palmitoyltransferase ZDHHC12	
80	Chrysin	7	P06493	0.655	Cyclin-dependent kinase 1	Jiang et al. [111]
95	Cryptotanshinone	1	P22303	0.473	Acetylcholinesterase	Obukhova et al. [98] and Lei et al. [102]
		7	P15559	0.362	NAD(P)H dehydrogenase quinone 1	

The name of the corroborated drug candidates, their predicted ranks based on consensus scoring ("Generating drug predictions and corroborating them using literature searches" section), the rank of the target from the top targets analysis ("Analyzing top targets and associated pathways for glioma" section), the target UniProt identifier, the predicted interaction score between these predictions and targets, the target name, and the evidence we found for the target being implicated in glioma, are listed. Higher scores indicate a higher likelihood of interaction. From this analysis, we highlighted Vitamin D3 receptor, thyroid hormone receptor, acetylcholinesterase, cyclin-dependent kinase 1, tubulin alpha chain, dihydrofolate reductase and thymidylate synthase as the most promising targets for glioma

## Discussion

The goal of this study was to evaluate the application of an established proteome-wide interaction–based platform in a disease-specific context, rather than to develop new cheminformatic or algorithmic methods. By focusing on glioma, we provide a systematic assessment of CANDO’s predictive performance and examine the biological relevance of its prioritized compounds. Such application-driven studies are complementary to methodological development efforts, as they help clarify the strengths, limitations, and translational utility of existing platforms in clinically relevant settings. Accordingly, the emphasis of this work is on benchmarking, disease-specific interpretation, and differentiation between rediscovered and novel candidates.

### Exploration of benchmarking results

The ligand-similarity (ECFP4/Tanimoto) pipeline modestly outperformed the proteomic signature pipeline in our benchmarking, and we applied a filter that suppressed the most extreme similarities within the 98th percentile to test whether these apparent successes are largely driven by very close chemical analogs. Near-duplicate "me too" compounds are common among approved drugs and late-stage investigational compounds because new drugs are often incremental structural variants of existing agents rather than entirely novel scaffolds [77–80]. The filter predictably attenuates performance in the all-indications benchmark because many indications contain tightly clustered drug classes where the highest-similarity neighbors are frequently same-indication drugs; removing those high-similarity edges strips away many of the within-class analog relationships that fingerprinting otherwise recovers near the top of each list. For example, Tanimoto nAIA drops from 11.300 for the unfiltered pipeline to 1.532 for the filtered pipeline at the Top 10 cutoff, and nNDCG drops from 0.065 to 0.008 at the same cutoff.

Despite the superiority of the unfiltered Tanimoto pipeline in *benchmarking*, the protein-centric approach remains valuable in *predictions* because it provides mechanistic interpretability that fingerprinting cannot: it explicitly models drug–protein interaction patterns that can be interrogated to generate hypotheses about targets, pathways, and modes of action. Ligand fingerprinting preferentially retrieves close structural neighbors, including “me too” compounds, while the proteomic signature method is more likely to surface chemically dissimilar, and therefore potentially more novel, repurposing candidates that converge on similar target sets and pathways. Prior work on CANDO highlights that structure-based pipelines remain valuable for precision medicine, where interaction scores can be recalculated under amino-acid variants to reflect patient-specific proteomes, and polypharmacy, where drug–proteome signatures can be integrated to propose rational combination therapies [77]. Both the fingerprinting and protein signature pipelines can be considered part of CANDO and represent different implementations of the same benchmarking workflow, since the CANDO platform is pipeline-agnostic.

Our data fusion results illustrate how integrating protein-centric and ligand-centric signals can yield better overall performance than either alone. By leveraging the accuracy and chemical neighborhood strength of fingerprinting while retaining the mechanistic and precision-medicine opportunities of protein-centric modeling, fusion synthesizes complementary information and mitigates the failures of individual pipelines. In this case, it reduces overreliance on close analogs in ligand space while mitigating the model-specific limitations and biases of a proteome-interaction scoring approach.

We next examined why the uniqueness filter behaves differently in glioma compared with the all-indications benchmark, and why that difference is less pronounced under the consensus-based metrics. In glioma, the filter is substantially less damaging to performance and can even be slightly beneficial at broader cutoffs. For example, Top 100 IA actually increases from 71.4 for the unfiltered Tanimoto pipeline to 74.2 for the filtered pipeline. Glioma’s known associations are relatively structurally diverse. We hypothesize that removing the top 2% of similarities therefore preferentially removes crowding by non-glioma-associated analogs at the very top of each glioma drug’s list, allowing other glioma drugs to surface into Top-N. The narrowing of the gap from all indications to glioma-specific metrics under the consensus framing (e.g. nIA, nNDCG) is less pronounced because the consensus list is not built from a single query drug’s nearest neighbors; instead, each approved drug contributes a binary vote for candidates that fall within its Top-N, and these votes are summed across drugs before ranking candidates by total support. This mechanism inherently dampens the crowding relief we observe in similarity-list benchmarking: removing a non-glioma analog from one glioma drug’s Top-N reduces that analog’s consensus support by one vote and may not be sufficient to remove it from the list entirely. The greater consistency of the consensus list metrics highlights their robustness and sophistication, as they are less sensitive to the idiosyncrasies of any single similarity list and more accurately represent the prediction protocol of CANDO. In contrast, the filtered Tanimoto pipeline’s higher Top 100 glioma IA reflects a metric-specific improvement that may not translate to better overall ranking performance, highlighting how the older metric can be unduly influenced by changes in a small handful of similarity lists. Previous work on CANDO has also noted the leniency of the IA metric relative to nIA [112].

### In depth analysis of corroborated drug candidates

CANDO identified potential glioma treatments that included drugs approved for other indications such as vitamin D (calcifediol), taxanes (cabazitaxel and docetaxel), vinca alkaloids (vinblastine and vinflunine), topoisomerase inhibitors (topotecan and irinotecan), and folic acid. Additionally, investigational compounds like ginsenosides, brivaracetam, chrysin, resiniferatoxin, and cryptotanshinone were also identified as promising drug candidates (Table 1). Literature-based analysis was conducted to corroborate these potential drugs and compounds for glioma, examining supporting evidence for their targets and pathways (Table 2 and Fig. 3). The top drug candidates generated via the interactomic signature pipeline of CANDO may be exerting their therapeutic effects by impacting multiple pathways implicated in glioma. We examined the corroborated drugs/compounds and targets predicted by CANDO in further detail, comparing and contrasting to what is known about their relevance to glioma in the literature. A detailed description follows below, beginning with compounds that are well-known in oncology based on our literature review and the volume of results we observed; these also serve as positive validation of our prediction pipeline.

Vitamin D3 analogs: Vitamins may have a role in the etiopathogenesis of central nervous system (CNS) cancers [113]. Vitamin D comprises a group of fat-soluble steroids, with vitamin D3 (cholecalciferol) and vitamin D2 (ergocalciferol) being the most significant [114]. Calcifediol (25-hydroxyvitamin D3),is the precursor for calcitriol, the active form of vitamin D [115]. Recent research suggests that the levels of the progenitor of calcitriol correlate with progression of glioma [116–119]. Cholecalciferol has shown promise in glioma treatment, especially glioblastoma multiforme (GBM), due to its ability to regulate cell cycle biomarkers and enhance the anti-tumor immune response [89, 120]. Studies indicate that vitamin D analogs, including ergocalciferol, could modulate biomarkers involved in cell cycle regulation and apoptosis in glioblastoma [120]. Cell cycle arrest is one of the most well-studied mechanisms accounting for the anti-tumor activity of vitamin D in gliomas. Vitamin D has been shown to induce anti-glioma effects through cell cycle arrest and the phosphoinositide 3-kinase (PI3K)/Akt pathway [91].

**Taxanes** are a class of diterpenes commonly used as chemotherapy agents, mainly including cabazitaxel, docetaxel and paclitaxel [121–123]. Cabazitaxel is a second-generation semisynthetic taxane. Contrary to other taxane compounds, cabazitaxel is a poor substrate for P-gp efflux pump; therefore, it easily penetrates across the BBB [124, 125]. Cabazitaxel shows a significant inhibitory effect on glioma [126, 127]. Other studies have reported that cabazitaxel exerts its anti-proliferative effects on cancer cells by binding to tubulin [128]. One study indicates that tubulin alpha-1C chain (TUBA1C) may potentially regulate the pathogenesis of glioma through Janus kinase (JAK)/signal transducer and activator of transcription (STAT) (JAK-STAT) pathway [129]. Docetaxel, a taxane-class anti-mitotic agent, demonstrates the ability to induce cell apoptosis in glioma and shows substantial inhibitory activity against tumor growth [130]. Furthermore, it is recognized as one of the leading drug candidates for brain tumor therapy [131]. In our study, both cabazitaxel and docetaxel are predicted to strongly interact with TUBA1C, with predicted interaction scores of 0.716 and 0.790, respectively (Table 2).

**Vinca alkaloids** are a class of chemotherapy agents with anti-mitotic and anti-microtubule properties, including compounds such as vinflunine, vinorelbine, vinblastine, and vincristine [132–134]. Vinflunine, a fluorinated vinca alkaloid, disrupts microtubule dynamics, a process essential for cell division, and has shown potential for glioma treatment [135, 136]. Vinorelbine, a semi-synthetic vinca alkaloid, is an anti-mitotic chemotherapy drug used to treat various cancers, including breast cancer, non-small cell lung cancer, and glioma [137]. Its antitumor effect arises from its ability to inhibit mitosis by interacting with tubulin [138]. In 2000, a pilot study of weekly vinblastine in patients with recurrent low-grade gliomas (LGG) yielded promising results [139, 140]. Compared to vinflunine and vinorelbine, vinblastine demonstrated a higher interaction score with the TUBA1C target (Table 2).

Topoisomerase I inhibitors: Topoisomerase inhibitors are chemical compounds that block the action of topoisomerases, which are broken into two broad subtypes: type I topoisomerases (TopI) and type II topoisomerases (TopII) [141, 142]. TopI inhibitors, like topotecan, are water-soluble camptothecin analogs that have shown cytotoxicity toward a variety of tumor types [143]. Topotecan can pass through the BBB and exhibits significant activity in treating glioblastoma multiforme [144, 145]. Additionally, it has been observed to induce cell cycle arrest at the G0/G1 and S phases [94, 146]. Irinotecan (CPT-11), a prodrug of 7-Ethyl-10-hydroxycamptothecin (SN-38), shows some antitumor activity in patients with recurrent glioblastoma multiforme, with response rates of 0–17% in several trials [147, 148]. Gimatecan is a lipophilic oral camptothecin analogue with preclinical activity in glioma models [149].

**Folic acid** (FA) targets the folate receptor (FR), which is overexpressed on the cell surface of various cancer cells [150–152]. Folate supplementation, particularly at high doses, has been suggested to have cytotoxic effects on glioma cells, making it a potential candidate for further exploration in glioma therapies [153]. In addition, utilizing lidocaine liposomes modified with folic acid has been demonstrated to suppress the proliferation and motility of glioma cells [154]. One clinical research study explored the role of folate-related compounds, such as L-methylfolate, in combination therapies for glioma, showing potential epigenetic modifications and enhanced sensitivity to standard treatments like temozolomide [155]. Zhao, et al. [105] hypothesized that inhibition of dihydrofolate reductase/thymidylate synthase might modulate the cell sensitivity of glioma cells to temozolomide through the mTOR signaling pathway. DHFR and TYMS are key metabolic enzymes in the folic acid signaling pathway, with high predicted interaction scores of 0.755 and 0.688, respectively, to folic acid in this study (Table 2).

**Ginsenosides**, active components found in Panax ginseng, show potential in glioma treatment due to their various therapeutic properties, including anticancer and neuroprotective effects [156, 157]. Additionally, ginsenoside has been shown to inhibit the growth of human glioma U251 cells, promoting apoptosis and affecting key signaling pathways involved in cell survival and death [158].

Compounds that are lesser known in oncology include the following. Several of these compounds, as noted earlier, are not yet approved and are therefore associated with a lower volume of published evidence. Moreover, the compounds in our candidate list (Table 1) that we do not discuss, lanosterol and ortataxel, due to limited supporting literature, are particularly worthy of experimental validation. Chrysin, an active natural bioflavonoid, is predicted to target cyclin-dependent kinase 1 with an interaction score of 0.655 (Table 2), and has been proven to protect against carcinogenesis [111]. Cyclin-dependent kinase is the target for glioma cell cycle arrest at G2 and M phases [159]. Chrysin exerts anticancer activity in glioblastoma cell lines possibly via the ERK/Nrf2 signaling pathway [160]. Resiniferatoxin, a naturally occurring irritant tricyclic diterpene which combines structural features of the phorbol ester tumor promoters and of capsaicin [161]. It activates transient vanilloid receptor (TRP), which was previously associated primarily with cardiovascular and neuronal regulation, but might also present avenues for exploration in glioma pathogenesis [162]. We observed evidence of interaction between resiniferatoxin and the thyroid hormone receptor beta target (Table 2 and Fig. 3). Cryptotanshinone is one of the main representative components isolated from the roots of Salvia miltiorrhiza. Lu et al. [163] indicated that cryptotanshinone can inhibit human glioma cell proliferation. Compounds such as brivaracetam, an alpha amino acid derivative, lack enzyme-inducing activity on the cytochrome system, and could be considered promising candidates for addressing brain tumor-related epilepsy [164, 165].

#### Predicted interactions with glioma markers and signaling modulators

As shown in Table 2 and Fig. 3, we predicted interactions between topotecan, irinotecan, and cryptotanshinone and acetylcholinesterase (AChE), a newly recognized marker for glioma (Table 2). One study reported that irinotecan or its metabolites directly interact with AChE, inhibiting the conversion of acetylcholine to choline, which leads to an accumulation of acetylcholine and subsequent cholinergic syndrome symptoms [166]. Bioinformatic analysis has shown that AChE is connected to proteins in the PI3K/Akt pathway, which promotes anti-apoptotic and proliferative effects in brain tumors [98]. There is limited evidence of interactions between topotecan, irinotecan, cryptotanshinone, and AChE; our study therefore provides predictive evidence of these interactions. Additionally, predictions of topotecan and resiniferatoxin targeting thyroid hormone receptor beta are novel to this study. The thyroid hormone receptor influences glioma progression by regulating the PI3K/Akt signaling pathway [96]. Therefore, candidates targeting the PI3K/Akt pathway may hold promise for glioma treatment.

### Absolute Jaccard coefficient in overlap analyses

The low Jaccard coefficients observed between CANDO-generated target sets and gold standard annotations arise in part from fundamental differences in how information is represented and used in these frameworks. CANDO compares compounds using full proteome-wide interaction signatures, where both strong interactions and weak scores are informative and contribute to similarity assessment by encoding non-interactions. In contrast, UniProt provides only positive associations and does not capture meaningful information about non-interactions. Applying a strict set-based overlap metric such as the Jaccard coefficient to these asymmetric representations is therefore inherently conservative and yields low absolute values, even when biologically relevant signal is present.

### Limitations

Our protein list, derived from predictions, highlights glioma-relevant targets but is inherently incomplete, similar to databases like UniProt or GeneCards, as each captures only a partial view of glioma biology. While this list serves as one of the curated gold standards for our analysis, incorporating known treatment targets in future studies could provide a more comprehensive benchmark. Limitations of this study include the arbitrary rank cutoffs, which may exclude moderately ranked targets that overlap meaningfully with gold standard libraries, and the use of the Jaccard coefficient, a binary metric that overlooks relative ranks or prediction scores. Additionally, our focus on glioma leaves the robustness of this approach across other indications underexplored, particularly for diseases with fewer validated targets. Finally, the analyses may bias toward frequently predicted top targets, underrepresenting less common targets with potential therapeutic value. To address these limitations, future studies will integrate score-based cutoffs, and consider a broader range of rank and score distributions.

### Future work

We acknowledge that the evidence supporting the 24 identified candidates is primarily derived from computational predictions and literature mining, which represents a limitation of the present study. Accordingly, this work is intended to be exploratory and hypothesis-generating in nature. Future studies may focus on experimental validation using established glioma cell lines to assess functional effects, as well as evaluation of blood–brain barrier permeability to further strengthen translational relevance. Another limitation of this study is that BBB permeability and ADME properties were not explicitly considered during candidate ranking. Consequently, some highly ranked compounds, such as taxanes and Vinca alkaloids, have limited clinical applicability in glioma due to poor BBB penetration and efflux liability. Importantly, candidate prioritization in CANDO is driven by proteome-wide interaction signature similarity and is therefore oriented towards finding therapeutically active compounds over pharmacokinetically optimized ones. Future work will incorporate BBB and ADME filtering as downstream steps to improve translational relevance. In this study, the selected fusion pipeline combined the proteomic and unfiltered Tanimoto approaches by multiplying their per-pair similarity scores. Future work could explore more sophisticated fusion strategies.

## Conclusions

We utilized our CANDO platform to explore potential novel treatments and their associated protein targets for glioma. By integrating a combination of computationally generated and experimentally observed data from benchmarking, prediction, corroboration of putative drug candidates using literature-based searches, top protein target analysis, and protein functional annotation, we identified promising treatments for glioma, including Vitamin D, taxanes, vinca alkaloids and topoisomerase inhibitors. Additionally, we highlighted several protein targets and related pathways linked to glioma, including Vitamin D3 receptor, thyroid hormone receptor, acetylcholinesterase, cyclin-dependent kinase 2, tubulin alpha chain, dihydrofolate reductase and thymidylate synthase. This study offers insights into the potential mechanisms underlying glioma and demonstrates the potential of the CANDO platform in identifying effective treatments against this disease.

## Supplementary Information

Supplementary material 1.

## Data Availability

CANDO is publicly available through Github at https://github.com/ram-compbio/CANDO. The code and data files are available https://doi.org/10.5061/dryad.g4f4qrg3j.

## Abbreviations

CANDO: Computational Analysis of Novel Drug Opportunities
BBB: Blood-brain barrier
P-gp: P-glycoprotein
ADME: Absorption, distribution, metabolism, and excretion
BANDOCK: Bioanalytical docking protocol
AF2: AlphaFold2
CTD: Comparative Toxicogenomics Database
MeSH: Medical Subject Headings
ECFP4: Extended Connectivity Fingerprints with a diameter of 4
RMSD: Root-mean-square deviation
IA: Indication accuracy
AIA: Average indication accuracy
nIA: New indication accuracy
NDCG: Normalized discounted cumulative gain
nNDCG: New NDCG
PI3K: Phosphatidylinositol-3’-kinase
mTOR: Mammalian target of rapamycin
JAK: Janus kinase
STAT: Signal transducer and activator of transcription
STAT3: Signal transducer and activator of transcription 3
AChE: Acetylcholinesterase
THRB: Thyroid hormone receptor beta
CDK1: Cyclin-dependent kinase 1
DHFR: Dihydrofolate reductase
TYMS: Thymidylate synthase
CNS: Central nervous system
GBM: Glioblastoma multiforme
TUBA1C: Tubulin alpha-1C chain
LGG: Low-grade gliomas
TOPI: Type I topoisomerases
TOPII: Type II topoisomerases
CPT-11: Irinotecan
SN-38: 7-Ethyl-10-hydroxycamptothecin
FA: Folic acid
FR: Folate receptor
TRP: Transient vanilloid receptor
NIH: National Institutes of Health
NCATS: NIH Clinical and Translational Sciences
NLM: NIH National Library of Medicine
NIST: National Institute of Standards of Technology
CCR: Center for Computational Research
